# Full alcohol marketing ban and adolescent drinking patterns: a repeated cross-sectional analysis comparing Lithuania with other EU countries

**DOI:** 10.1136/bmjph-2025-004245

**Published:** 2026-04-10

**Authors:** Daniela Correia, Jakob Manthey, Anastasia Månsson, Peter Allebeck, Ludwig Kraus, Jürgen Rehm

**Affiliations:** 1 Department of Mathematics, Faculty of Sciences, University of Porto, Porto, Portugal; 2 EPIUnit—Instituto de Saúde Pública, Universidade do Porto, Porto, Portugal; 3 Laboratório para a Investigação Integrativa e Translacional em Saúde Populacional, Porto, Portugal; 4 Centre for Interdisciplinary Addiction Research (ZIS), Department of Psychiatry and Psychotherapy, University Medical Center Hamburg-Eppendorf, Hamburg, Germany; 5 Department of Psychiatry, University of Leipzig Faculty of Medicine, Leipzig, Germany; 6 Department of Global Public Health, Karolinska Institute, Stockholm, Sweden; 7 Department of Public Health Sciences, Centre for Social Research on Alcohol and Drugs, Stockholm University, Stockholm, Sweden; 8 Institute of Psychology, ELTE Eötvös Loránd University, Budapest, Hungary; 9 Institute for Mental Health Policy Research, Centre for Addiction and Mental Health (CAMH), Toronto, Ontario, Canada; 10 Campbell Family Mental Health Research Institute, Centre for Addiction and Mental Health (CAMH), Toronto, Ontario, Canada; 11 Dalla Lana School of Public Health, University of Toronto, Toronto, Ontario, Canada; 12 Program on Substance Abuse & WHO European Region Collaboration Centre, Public Health Agency of Catalonia, Barcelona, Spain; 13 World Health Organization/Pan American Health Organization Collaborating Centre, Centre for Addiction and Mental Health, Toronto, Ontario, Canada; 14 Institute of Medical Science, Temerty Faculty of Medicine, Toronto, Ontario, Canada

**Keywords:** Public Health, Adolescent, trends

## Abstract

**Introduction:**

Alcohol consumption is a leading cause of premature mortality in Europe, with adolescents particularly vulnerable to marketing influences. While alcohol marketing bans are considered a WHO ‘best buy’, evidence of their effectiveness remains limited. Lithuania’s 2018 full national ban on alcohol marketing offers a unique opportunity to assess its impact on adolescent drinking. This study evaluated whether adolescents in Lithuania reported greater declines in alcohol use compared with peers in European Union countries without a full ban.

**Methods:**

We conducted a difference-in-difference analysis using generalised linear regression models to assess alcohol use among adolescents. We used data from the European School Survey Project on Alcohol and Other Drugs for 15–16 year-olds in Lithuania and five comparator countries (Estonia, France, Italy, Latvia and Poland) across four waves (2007, 2011, 2015 and 2019; n=84 189). The study follows a prespecified and published protocol. The primary outcome was self-reported frequency of intoxication in the past 12 months, assessed by negative binomial regression models adjusted for individual and country-level covariates.

**Results:**

Compared with statutory control for some alcoholic beverages, a full marketing ban was associated with a 35% reduction in frequency of intoxication (incidence rate ratios (IRR) 0.65 (95% CI 0.56 to 0.77)). Sensitivity analyses confirmed this association. Without the ban, predicted intoxication frequency in 2019 would have been nearly twice as high (1.28 vs 0.73 occasions). Additional analyses showed significant reductions in the odds of any intoxication (OR 0.68 (95% CI 0.60 to 0.78)). Protective associations were also observed for alcohol use (IRR 0.88 (95% CI 0.79 to 0.99)) and binge drinking (IRR 0.82 (95% CI 0.72 to 0.92)).

**Conclusions:**

The implementation of full alcohol marketing bans was associated with reduced risky drinking behaviours among adolescents in Lithuania. Our findings support full bans as a central component of alcohol control, especially in the digital age, where partial restrictions are easily circumvented.

**WHAT IS ALREADY KNOWN ON THIS TOPIC**

- Alcohol marketing exposure is strongly linked to early initiation and risky drinking among adolescents, and the WHO recommends marketing bans as a ‘best buy’ for alcohol control. However, real-world evidence on the effectiveness of full national bans remains scarce.

**WHAT THIS STUDY ADDS**

- This study provides empirical evidence that a full alcohol marketing ban in Lithuania was associated with significant reductions in adolescent intoxication and other risky drinking behaviours, beyond secular trends and other policy measures.

**HOW THIS STUDY MIGHT AFFECT RESEARCH, PRACTICE OR POLICY**

- Full marketing bans should be considered a key component of alcohol control strategies, particularly for young people in the digital era, where partial restrictions are easily circumvented, and highlight the need for further evaluations in diverse settings.

## Introduction

 Alcohol consumption remains a leading cause of premature mortality and disease burden in the WHO European Region, accounting for one in every 10 deaths overall, and an even greater share among young people.^1^ In Europe, alcohol use often begins early, with one in three adolescents reporting having their first drink by age 13.^2^ As young people progress through adolescence and into young adulthood, consumption typically increases during a period when they are particularly susceptible to external influences such as social networks and advertisements for alcohol.^3^

Given its substantial contribution to morbidity and mortality, reducing alcohol consumption has become a global public health priority. The WHO’s 2013 global action plan for the prevention and control of non-communicable diseases identified three ‘best buys’ for alcohol control: increasing alcohol taxes, restricting availability, and banning alcohol marketing and advertising.^4^ Among the three best buys for alcohol, marketing bans remain the most scientifically contested policy. There is substantial evidence linking alcohol marketing to drinking intentions, consumption and harmful drinking behaviours,^5^ and this association is particularly strong among youth. Meta-analyses and longitudinal studies consistently show that exposure to alcohol marketing increases the likelihood of early initiation and binge drinking in youth,^3 6^ and recent reviews conclude that the relationship is likely causal.^7^ Yet, a 2014 Cochrane Review and a more recent literature review identified insufficient evidence for the effectiveness of alcohol marketing bans in reducing population alcohol use.^8 9^ Despite ongoing debate around the role of marketing in shaping alcohol use, real-world evaluations of full marketing bans remain limited. Given its status as a ‘best buy’, alcohol marketing bans deserve more rigorous evaluation—particularly in populations in which effects are most likely to be observed. Adolescents represent such a population, as they are especially susceptible to alcohol marketing and disproportionately exposed, particularly online,^10^ offering the clearest window into the potential effects of alcohol marketing bans.^11^

Lithuania offers a rare opportunity to evaluate a full alcohol marketing ban in a real-world setting in a high-income country. Introduced in 2018 as part of an integrated package of alcohol control reforms, the policy prohibits nearly all forms of alcohol marketing, including digital and social media. The ban was enacted in response to high levels of alcohol-related harm, after Lithuania was reported to have had the highest *per capita* alcohol consumption in the European Union (EU) in 2016.^12^ It remains the only EU country to have fully implemented all three WHO ‘best buys’ in a short period, and the only high-income country in more than three decades to adopt a full national alcohol marketing ban. Previous examples of marketing bans among high-income countries include Norway, which has maintained a full ban on alcohol marketing since 1975, covering all beverages and all types of media, including digital platforms.^13^ Finland had an almost full ban from 1977 until 1994, when it was partially lifted as part of policy liberalisation in preparation for EU accession, allowing advertising for beverages up to 22% alcohol by volume.^14^ As such, Lithuania provides a unique natural experiment for assessing the impact of such policies on youth drinking.

Aiming to assess the impact of Lithuania’s 2018 marketing ban on youth drinking, we hypothesised that its implementation would be followed by reductions in adolescent alcohol use, particularly intoxication, as prespecified in a published study protocol.^15^ Given the general trend of declining youth alcohol use in Europe,^2^ this analysis investigates whether adolescents in Lithuania reported greater declines in alcohol use compared with their peers in other EU countries where no full marketing ban was implemented.

## Methods

### Study design

We conducted a difference-in-difference analysis using generalised linear models to assess alcohol use among 15–16-year-old adolescents, using data from repeated cross-sectional surveys conducted in 2007, 2011, 2015 and 2019. The study included Lithuania and five comparator countries (Estonia, France, Italy, Latvia and Poland), selected to reflect a range of alcohol policy environments, particularly regarding marketing restrictions and other population-level measures. This study follows a prespecified and published protocol.^15^

### Policy setting and country selection

Lithuania implemented a full alcohol marketing ban effective 1 January 2018. The ban covers advertising across all media types and promotional activities such as games and competitions. As a result, alcohol advertising has disappeared from television, radio, digital media and Lithuanian-language print media, including materials produced by foreign publishers targeting the national market. Some residual marketing channels remain outside the scope of the ban, including marketing of non-alcoholic beverages produced by the same companies using similar branding, and cross-border advertising such as alcohol promotions in foreign print magazines that may still circulate domestically.^12^ Enforcement of the statutory ban has been active. On social media platforms, the Drug, Tobacco and Alcohol Control Department monitors compliance, with authority to block non-compliant websites and impose civil penalties, while digital platforms conduct automated ad reviews aligned with national regulations. Compliance has been consistently high, with alcohol advertising accounting for less than 5% of infringements annually and only 1.4% of monitored social media posts violating the ban.^16^

Lithuania’s marketing ban was part of a broader alcohol control package to address the country’s high levels of alcohol-related harm. Additional measures introduced on the same day included reduced retail hours for off-premises alcohol sales and increasing the minimum legal purchasing age from 18 to 20 years. These changes followed an excise tax increase implemented in March of 2017.

The five comparison countries, Estonia, Latvia, Poland, France and Italy, were selected based on geographical proximity, similarities in drinking patterns or the implementation of other key alcohol control policies. Estonia and Latvia introduced partial marketing restrictions, time-based availability limits and excise tax increases. Poland implemented fewer control measures, having eased some alcohol policies in the early 2000s, followed by some moderate tax adjustments. France and Italy raised the minimum legal purchasing age from 16 to 18 and implemented additional measures such as blood alcohol concentration (BAC) limits for novice drivers, excise tax changes and youth-targeted advertising restrictions. Further details on policy timing and content are available in online supplemental appendix 1.

### Data sources

Alcohol policy data, including marketing restrictions and other control measures, were drawn from a longitudinal dataset compiled by Månsson and colleagues covering the years 2000–2019.^17 18^ These data were gathered as part of a project evaluating alcohol policies across European countries and over time, using the modified Bridging the Gap (BtG-M) scale: control of production and wholesale of alcohol, control of distribution, personal control (ie, legal age limits for purchases), control of marketing, social and environmental controls (ie, BAC limits for driving), public policy, and alcohol taxation.^19^ The version of the BtG-M scale used in this study was obtained from the authors and reflects the developed scale as of August 2025.

Youth alcohol use data were obtained from the European School Survey Project on Alcohol and Other Drugs (ESPAD),^2^ a repeated cross-national, school-based survey of 15- and 16-year-olds. The survey, conducted every 4 years since 1995, provides comparable data on substance use and related behaviours across European countries.

Five waves of data collection were available for all six countries in this study at the time of data analysis: 2003, 2007, 2011, 2015 and 2019. However, the 2003 wave was excluded due to a change in the question format for intoxication, which broke comparability with later waves, as confirmed by a split-half reliability test conducted in 2007 (results not shown).

### Outcome variables

The primary outcome was self-reported frequency of intoxication in the past 12 months, as per protocol, assessed with the question: ‘on how many occasions (if any) have you been intoxicated from drinking alcoholic beverages, for example, staggered when walking, not being able to speak properly, throwing up or not remembering what happened?’. Response options were: 0, 1–2, 3–5, 6–9, 10–19, 20–39, or 40 or more occasions. Responses were converted to counts using the midpoint of each interval category.

Secondary outcomes were self-reported frequency of alcohol consumption in the past 12 months (0, 1–2, 3–5, 6–9, 10–19, 20–39, or 40 or more occasions) and self-reported frequency of binge drinking (five or more alcoholic drinks on a single occasion) in the past 30 days (0, 1, 2, 3–5, 6–9, or 10 or more times), also treated as a count variable.

### Independent variables

Country-level alcohol policy restrictiveness was assessed using the seven BtG-M domains,^17^ reflecting policies in place at each survey year, and modelled as year-dependent covariates. The marketing control score, our primary exposure, was scored as follows: no national restrictions (0 pt); statutory control on national alcohol advertising for some alcoholic beverages (1 pt); ban on national alcohol advertising on some types of advertising media (1.5 pt); ban on national alcohol advertising for some types of alcoholic beverages (2 pt) and ban on all national alcohol advertising and sponsorship (3 pt). In our sample, the score ranged between 1 pt (Italy, in 2007, 2011, 2015 and 2019) and 3 pt (Lithuania, in 2019). The scoring scheme of the other domains is detailed in online supplemental appendix 2. Each domain was treated as continuous and rescaled from 0 (least) to 1 (most restrictive) before analysis.

Individual-level data from the ESPAD included sex (male or female), social behaviours and self-perceived availability of alcoholic beverages.^2^ Social behaviours—sports participation, going out in the evening, spending time with friends and internet use—were measured on a five-point scale and categorised as less than once or twice a month, or at least once a week. Perceived availability of beer, wine and spirits was measured on a five-point scale from ‘impossible’ to ‘very easy’, categorised as ‘impossible to fairly difficult’, or ‘fairly to very easy’.

### Final sample

The 2007, 2011, 2015 and 2019 waves of the ESPAD for the six countries selected comprised 85 548 adolescents.^2^ Of these, 84 189 had measured data on the primary outcome and were included in the final sample.

### Statistical analysis

Negative binomial regression was used to model the mean number of intoxication occasions (frequency) in the past 12 months and its association with alcohol marketing restrictions. This distribution was selected after testing alternatives within the exponential family, including zero-inflated models, due to the high proportion of non-drinkers. Results are reported as incidence rate ratios (IRR) with 95% CIs.

Individual-level and time-varying country-level covariates were tested and retained if significant, following a blockwise approach: (1) year and country, (2) sex, (3) social behaviours, (4) perceived alcohol availability and (5) alcohol policy restrictiveness. Of the seven BtG-M domains measuring alcohol policy restrictiveness, control of production was excluded due to limited variability, and social and environmental controls for collinearity with the country.

The same modelling strategy was applied to secondary outcomes: frequency of alcohol consumption (12-month recall) and frequency of binge drinking (past 30 days).

As additional analysis, we ran a two-part model for the primary outcome: (1) logistic regression for the prevalence of intoxication in the past 12 months, and (2) negative binomial regression for the frequency of intoxication among those reporting at least one episode of intoxication in the past 12 months. Both models were adjusted for significant covariates as described before. We further tested model robustness by excluding the personal control domain from the main model. Finally, we conducted a sensitivity analysis using a binary indicator for a full national advertising and sponsorship ban instead of the continuous BtG-M marketing control domain, ie, ‘0’ for no or partial ban on marketing (scores 0–2 pt), and ‘1’ for a full ban on marketing (score 3 pt—only observed in Lithuania in 2019).

Finally, we conducted a power analysis for the main outcome, assessing whether the observed effect size was detectable given the sample size; details and results are provided in online supplemental appendix 7.

All analyses were conducted in R (V.4.3.3) with a significance threshold of 0.05.

## Results

The number of participants included in the analytic sample (n=84 189) by country and data collection wave is shown in table 1. The time-varying country-level BtG-M domain scores are shown in online supplemental appendix 2, and individual-level sample characteristics are detailed in online supplemental appendix 3.

**Table 1:** Sample size included in the analysis, by country and ESPAD data collection wave

Country	2007	2011	2015	2019	Total
Estonia	2330 (10.8%)	2413 (11.7%)	2419 (9.9%)	2478 (14.1%)	9640 (11.5%)
France	2882 (13.3%)	2549 (12.4%)	2674 (11%)	2539 (14.4%)	10 644 (12.6%)
Italy	9762 (45.1%)	4807 (23.3%)	4016 (16.5%)	2510 (14.3%)	21 095 (25.1%)
Latvia	2208 (10.2%)	2559 (12.4%)	1044 (4.3%)	2689 (15.3%)	8500 (10.1%)
Lithuania	2345 (10.8%)	2405 (11.7%)	2518 (10.3%)	2372 (13.5%)	9640 (11.5%)
Poland	2097 (9.7%)	5872 (28.5%)	11 702 (48%)	4999 (28.4%)	24 670 (29.3%)

ESPAD, European School Survey Project on Alcohol and Other Drugs.

**Figure 1:**
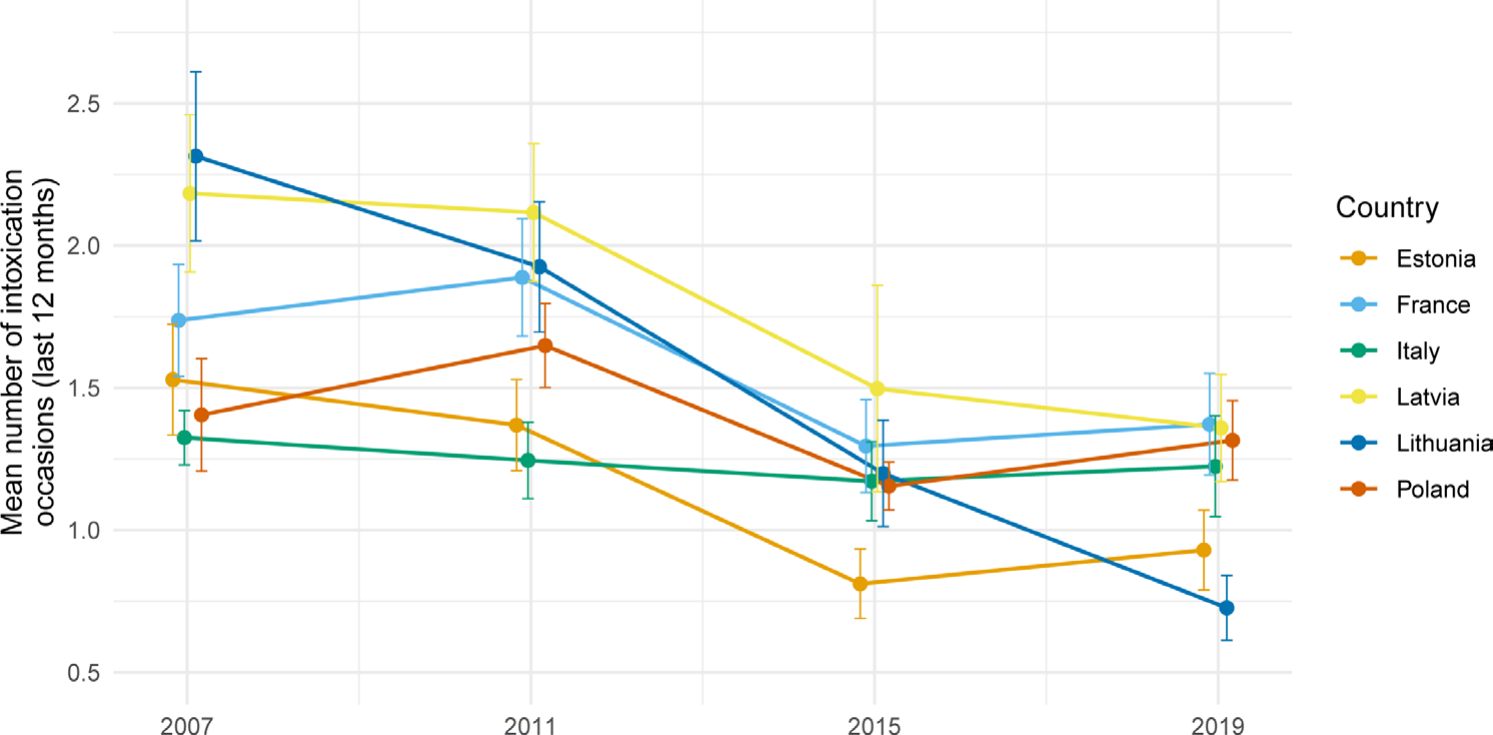
Observed mean frequency of adolescent intoxication in the past 12 months, by country and European School Survey Project on Alcohol and Other Drugs data collection wave. Note: error bars represent 95% CIs for the mean.

Figure 1 illustrates changes in the frequency of intoxication among adolescents across the six countries over the four survey waves (see also online supplemental appendix 3). Overall, levels remained relatively stable between 2007 and 2011, followed by a marked decline between 2011 and 2015, with this lower level being maintained in 2019. In 2007, Lithuania had the highest average number of intoxication occasions in the last 12 months (2.31 (95% CI 2.21 to 2.43)), and by 2019, following the implementation of its alcohol marketing ban, the lowest (0.73 (95% CI 0.69 to 0.76)).

Table 2 presents the adjusted regression models for the primary outcome of this study. After accounting for secular trends and individual- and country-level covariates, a complete alcohol marketing ban was significantly associated with lower frequency of intoxication (IRR 0.65 (95% CI 0.56 to 0.77)). This means that, compared with a less restrictive statutory control for some alcoholic beverages, the full ban implemented in Lithuania in 2018 was associated with a 35% reduction in the mean frequency of intoxication among adolescents in 2019. In a sensitivity analysis measuring marketing control as a dichotomic variable for a full ban, we also found a 44% reduction in the frequency of intoxication among adolescents (IRR 0.56 (95% CI 0.46 to 0.67), see online supplemental appendix 5).

**Table 2:** Adjusted regression models assessing adolescent intoxication in the past 12 months

	Frequency of intoxication (n=84 189)	Two-part model	
		Prevalence of intoxication (n=84 189)	Frequency of intoxication among those reporting intoxication (n=26 267)
	IRR (95% CI)*	OR (95% CI)†	IRR (95% CI)*
*BtG-M scores*			
Control of distribution	–	–	0.84 (0.75 to 0.94)
Personal control	1.64 (1.45 to 1.85)	1.79 (1.59 to 2.01)	1.11 (1.01 to 1.22)
Control of marketing	0.65 (0.56 to 0.77)	0.68 (0.60 to 0.78)	0.88 (0.75 to 1.03)‡
Public policy	–	0.88 (0.82 to 0.94)	–
Alcohol taxation	0.61 (0.49 to 0.77)	–	0.71 (0.59 to 0.87)
Country			
Lithuania	Ref	Ref	Ref
Estonia	0.92 (0.85 to 1.00)	1.02 (0.94 to 1.10)	0.84 (0.79 to 0.89)
France	1.20 (1.09 to 1.31)	1.45 (1.33 to 1.57)	1.04 (0.96 to 1.13)
Italy	0.52 (0.46 to 0.59)	0.49 (0.44 to 0.54)	0.85 (0.77 to 0.93)
Latvia	1.34 (1.23 to 1.46)	1.33 (1.23 to 1.45)	1.07 (0.97 to 1.17)
Poland	0.84 (0.76 to 0.92)	0.74 (0.69 to 0.80)	0.99 (0.89 to 1.11)
ESPAD collection wave			
2007	Ref	Ref	Ref
2011	1.03 (0.97 to 1.09)	0.97 (0.91 to 1.02)	1.04 (0.99 to 1.10)
2015	0.79 (0.73 to 0.85)	0.74 (0.69 to 0.78)	0.98 (0.92 to 1.05)
2019	0.87 (0.79 to 0.97)	0.69 (0.65 to 0.74)	1.10 (1.01 to 1.20)
Sex			
Male	Ref	Ref	Ref
Female	0.69 (0.67 to 0.72)	0.91 (0.88 to 0.95)	0.73 (0.71 to 0.75)
*Social behaviours*			
Sports participation			
Less than once or twice a month	Ref	Ref	Ref
At least once a week	0.71 (0.68 to 0.74)	0.81 (0.78 to 0.85)	0.83 (0.80 to 0.86)
Going out in the evening			
Less than once or twice a month	Ref	Ref	Ref
At least once a week	2.35 (2.26 to 2.44)	2.24 (2.15 to 2.32)	1.45 (1.41 to 1.49)
Hanging out with friends			
Less than once or twice a month	Ref	Ref	Ref
At least once a week	1.50 (1.44 to 1.56)	1.57 (1.51 to 1.63)	1.10 (1.07 to 1.14)
Using the internet for leisure			
Less than once or twice a month	Ref	Ref	Ref
At least once a week	0.90 (0.84 to 0.95)	1.12 (1.05 to 1.19)	0.88 (0.84 to 0.92)
*Access to alcoholic beverages*			
Beer			
Impossible or very or fairly difficult	Ref	Ref	Ref
Fairly or very easy	1.51 (1.42 to 1.61)	1.91 (1.79 to 2.05)	0.92 (0.86 to 0.98)
Wine			
Impossible or very or fairly difficult	Ref	Ref	Ref
Fairly or very easy	1.16 (1.09 to 1.22)	1.10 (1.04 to 1.17)	1.08 (1.03 to 1.14)
Spirits			
Impossible or very or fairly difficult	Ref	Ref	Ref
Fairly or very easy	2.73 (2.60 to 2.87)	2.48 (2.36 to 2.60)	1.47 (1.41 to 1.54)
Country			
Lithuania	Ref	Ref	Ref
Estonia	0.92 (0.85 to 1.00)	1.02 (0.94 to 1.10)	0.84 (0.79 to 0.89)
France	1.20 (1.09 to 1.31)	1.45 (1.33 to 1.57)	1.04 (0.96 to 1.13)
Italy	0.52 (0.46 to 0.59)	0.49 (0.44 to 0.54)	0.85 (0.77 to 0.93)
Latvia	1.34 (1.23 to 1.46)	1.33 (1.23 to 1.45)	1.07 (0.97 to 1.17)
Poland	0.84 (0.76 to 0.92)	0.74 (0.69 to 0.80)	0.99 (0.89 to 1.11)

Note: the primary outcome of this study was frequency of intoxication (shaded in grey); the other two models were included as post hoc analyses.

* IRR and respective 95% CIs, obtained from adjusted negative binomial regression models.

† OR and respective 95% CIs, obtained from the adjusted logistic regression model.

‡ Control of marketing, the main exposure of the study, was not significant but still included in the model.

BtG-M, modified Bridging the Gap scale; ESPAD, European School Survey Project on Alcohol and Other Drugs; IRR, incidence rate ratios.

**Figure 2:**
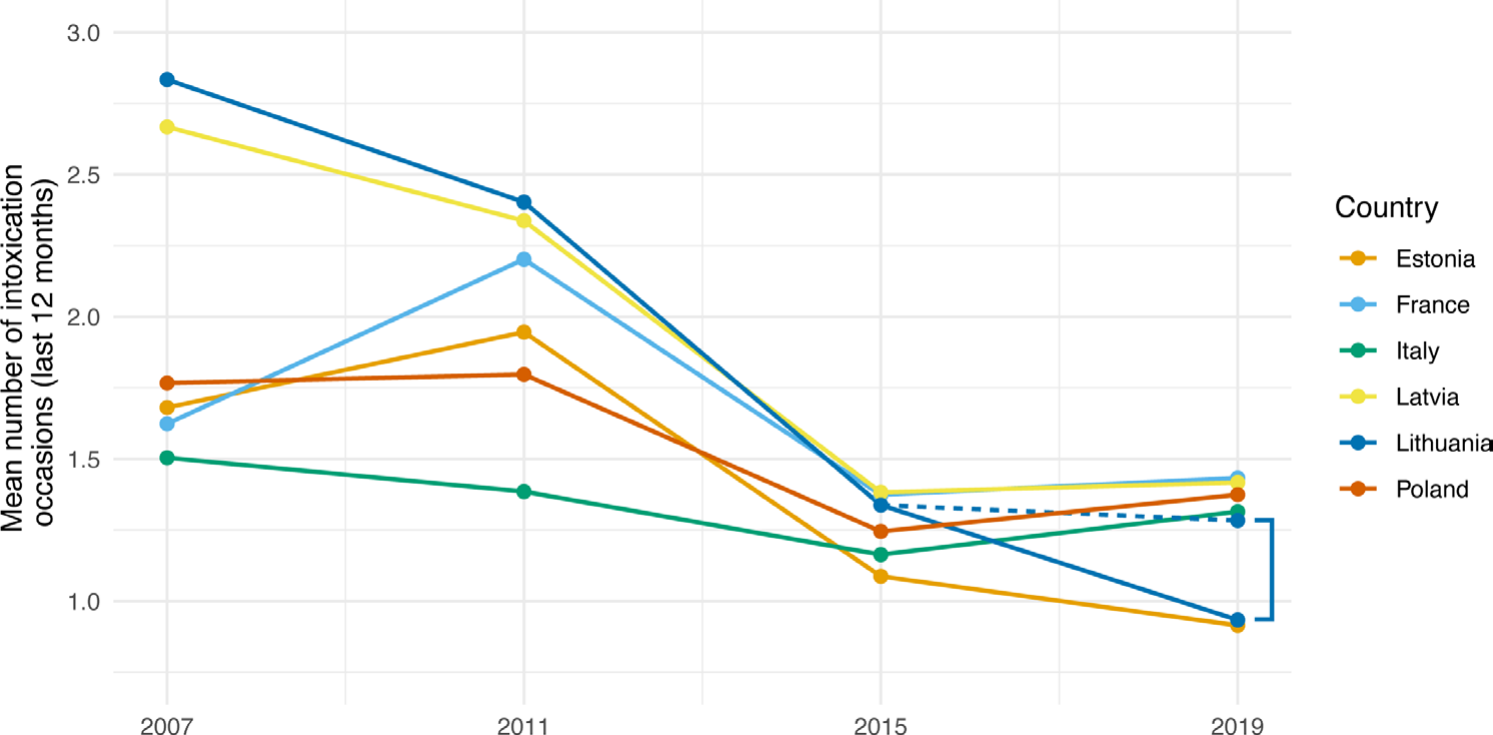
Estimated impact of Lithuania’s 2018 alcohol marketing ban on mean frequency of adolescent intoxication, based on European School Survey Project on Alcohol and Other Drugs data. Note: full lines represent the modelled values by the adjusted negative binomial regression model. The dashed line represents the predicted value for Lithuania if the 2018 alcohol marketing ban had not been implemented while keeping all other policy changes in effect. The square bracket indicates the impact of Lithuania’s 2018 alcohol marketing ban on the average frequency of intoxication.

The impact of Lithuania’s 2018 alcohol marketing ban on the mean frequency of intoxication of adolescents is shown in figure 2. Had the ban not been implemented—while retaining all other policies in effect at that time—the predicted mean frequency of intoxication in 2019 in Lithuania would have been 1.31 (95% CI 1.21 to 1.39), almost 40% higher than the observed level (0.94 (95% CI 0.87 to 1.00)).

The additional two-part model analysis shows two patterns (see table 2; see also online supplemental appendix 3): a significant reduction in the odds of reporting any intoxication (OR 0.68 (95% CI 0.60 to 0.78); corresponding to a relative risk 0.75 (95% CI 0.68 to 0.83))^20^ and a tendency toward lower frequency among those who reported at least one intoxication episode (IRR 0.88 (95% CI 0.75 to 1.03)), although this latter association did not reach statistical significance. Similar to the mean frequency of intoxication, had the ban not been implemented—while retaining all other policies in effect at that time—the predicted prevalence of intoxication in 2019 in Lithuania would have been 32.9% (95% CI 0.313% to 0.344%), instead of the observed 27.8% (95% CI 0.260% to 0.297%).

The association with most individual- and country-level covariates followed anticipated patterns (see table 2): a secular decline in frequency of intoxication was observed between 2011 and 2015, females reported lower frequency than males, and sports participation and internet use showed associations with reduced intoxication. Frequent socialising and easier access to alcohol, particularly spirits, were associated with a higher frequency of intoxication. Among policy measures, higher alcohol taxation was associated with lower frequency of intoxication, while personal control measures showed an unexpected positive association, that is, a lower level of control associated with higher frequency of intoxication. To account for this unexpected result, we tested a more conservative model excluding the personal control domain (see online supplemental appendix 5). The association between marketing restrictions and frequency of intoxication decreased but remained significant (IRR 0.79 (95% CI 0.68 to 0.92)), reinforcing the independent effect of marketing control.

The associations of marketing restrictions with secondary outcomes are shown in online supplemental appendix 6. Similarly to the result for frequency of intoxication, marketing control was significantly associated with both the secondary outcomes analysed, but showed a smaller effect size— higher marketing restrictions were associated with a 12% reduction in frequency of alcohol consumption in the past 12 months (IRR 0.88 (95% CI 0.79 to 0.99)), and 18% reduction in frequency of binge drinking in the past 30 days (IRR 0.82 (95% CI 0.72 to 0.92)).

## Discussion

This study provides real-world evidence that a full national ban on alcohol marketing can reduce risky drinking behaviours among adolescents. To our knowledge, this is one of the few studies that have empirically demonstrated such an impact. Following Lithuania’s 2018 ban, we observed a large decline in the prevalence and frequency of alcohol intoxication among adolescents protected by this ban, above and beyond secular trends and the association with other alcohol control measures in place at the time. The findings suggest that without the marketing ban, Lithuania would not have recorded the lowest intoxication rates among the six countries in 2019, despite broader alcohol policy efforts.

Cross-country trends also help contextualise the observed changes. Lithuania showed a steeper decline in 2007–2011 than the other countries, but this period coincided with the introduction of strong alcohol control measures in 2008–2009 rather than reflecting a distinct secular trend. Between 2011 and 2015, when Lithuania did not introduce major new alcohol control measures, the country displayed a pattern similar to the broader European trend of declining intoxication (see online supplemental appendix 4). In 2015–2019, a new comprehensive set of alcohol control policies was introduced in Lithuania, which explains the renewed steep decline compared with the other countries. In contrast, Italy consistently exhibited comparatively low levels of reported intoxication. This aligns with Southern European drinking cultures, particularly in predominantly Catholic contexts, where alcohol is integrated into meals and visible drunkenness is strongly discouraged.^21^ These cultural norms shape both adolescents’ behaviour and the likelihood of reporting intoxication in surveys. By comparison, Lithuania reflects a Northern and Eastern European drinking culture in which episodic heavy drinking and visible intoxication have historically been more socially accepted.

The impact of Lithuania’s marketing ban appears to have been immediate, aligning with evidence that short-term policy impacts are key in public health decision-making.^22^ The ban took effect in January 2018, and its impact was visible in the 2019 ESPAD data. Given the 12-month recall period, this reflects the behaviour of adolescents protected by the ban for just over a year, and thus may represent an early, not yet stabilised policy effect, which may strengthen or attenuate over time. However, while this short-term reduction is encouraging, the longer-term implications may be even more far-reaching, as future cohorts who grow up with little to no exposure to alcohol marketing may develop entirely different norms and expectations around alcohol.

The association between marketing control and reduced alcohol use was observed for both frequency and prevalence of intoxication, as well as frequency of consumption in the past 12 months, or binge drinking in the past 30 days. These findings suggest that marketing bans influence adolescents’ overall drinking patterns, in particular making them less likely to drink to intoxication under stricter marketing regulations. This could reflect how digital alcohol marketing, driven by algorithmic targeting, reinforces existing behaviours, potentially increasing heavy drinking.^23^ Marketing bans may therefore be more effective in reducing the severity of risky drinking patterns than in lowering overall drinking prevalence or frequency.

Finally, the findings underscore that full marketing bans that prohibit all national alcohol advertising and sponsorship are considerably more effective than partial marketing restrictions. This is not surprising given the limitations of partial marketing restrictions. History shows that anything short of a full ban often leads to a shift in marketing to unregulated channels, platforms or time slots, creating an adaptive environment where exposure persists or even increases. For example, when the Netherlands restricted alcohol advertising to late-night hours in 2010, exposure dropped among young children but increased among high-risk adolescents.^24^

### Limitations

This study has several limitations. All alcohol-related outcomes were based on self-reported survey data, and while this is standard in adolescent substance use research, it may be affected by under-reporting or recall bias. However, validation studies of the ESPAD questionnaire suggest it yields reasonably reliable and comparable estimates across countries.^25^ There may also be cultural differences in how adolescents interpret and report intoxication that could introduce bias, though such perceptions are likely stable over time and thus unlikely to explain observed changes. Moreover, although we adjusted for a range of individual- and country-level covariates, the ecological design limits causal inference.

The BtG-M scale captures the existence of national-level policies but does not reflect differences in enforcement or implementation, as its binary coding may miss variation in policy intensity (eg, a 1-hour vs 6-hour restriction on sales is treated equally), and it does not reflect subnational regulations. We also did not account for the exact timing of policy implementation relative to data collection, which may influence observed effects. In addition, policy effects may be amplified when introduced as part of a broader package, as in Lithuania, so the observed associations with marketing may partly reflect interaction effects between alcohol policies that could not be tested and thus should be interpreted in that context.

Another potential limitation concerns the number of countries included in the analysis. Although previous cross-national studies have used a larger number of countries (eg,^11^ our selection was constrained by the availability of comparable, detailed and longitudinal policy data from the BtG-M scale. Moreover, we purposively selected countries that implemented at least one major alcohol control change during the study period to maximise meaningful policy contrasts rather than sample size.^26^ Nonetheless, the limited number of countries may introduce concerns about selection bias and reduce the generalisability of the findings. Future research should replicate these analyses with broader country samples as more comprehensive policy datasets become available.

### Marketing ban versus other policies

Among the alcohol policy measures analysed, marketing restrictions showed the second strongest negative association with adolescent frequency of intoxication. Alcohol taxation was also significantly associated with lower intoxication, aligning with evidence that price strongly influences alcohol use. In the context of the six countries studied, the highest level of taxation had a slightly greater impact on reducing adolescent intoxication than the highest level of marketing restrictions. Both policies remained independently significant when mutually adjusted, suggesting distinct behavioural mechanisms. For adolescents, this implies that taxation alone may not offer the same protection as full marketing bans.

An unexpected positive association was observed between personal control measures, such as the minimum legal purchasing age, and frequency of intoxication. This likely reflects implementation timing in countries such as France or Italy, where such policies were introduced later and in isolation, possibly in response to rising adolescent drinking. In this context, personal control policies may be a consequence of, rather than the cause of, higher intoxication. This may have led to some overestimation of the marketing control effect size, though the association remained strong even in the additional model that excluded personal control.

## Conclusion

While partial restrictions appear insufficient, full marketing bans are associated with shifts in youth drinking patterns away from high-risk behaviours. These associations were observed within a year of implementation and may strengthen over time as younger cohorts grow up with little or no exposure to alcohol marketing. Full bans should therefore be considered a key component of alcohol control strategies, complementing measures such as taxation to better protect young people from alcohol-related harm. As more countries seek to reduce alcohol-related harm, Lithuania’s experience offers a compelling case for adopting stronger, enforceable marketing regulations to protect the next generation.

## Supplementary material

10.1136/bmjph-2025-004245 online supplemental file 1

## Data Availability

Data may be obtained from a third party and are not publicly available.
