# Supplementary material for: Full alcohol marketing ban and adolescent drinking patterns: a repeated cross-sectional analysis comparing Lithuania with other EU countries
Source: BMJ Public Health. 2026 Apr 10;4(2):e004245. doi: 10.1136/bmjph-2025-004245 (PMC13084893; doi:10.1136/bmjph-2025-004245)
Supplement: online supplemental file 1 [file bmjph-4-2-s001.docx]

**Appendices**

**Title: A full alcohol marketing ban and adolescent drinking patterns: a repeated cross-sectional analysis comparing Lithuania with other EU countries**

**Authors:** Daniela Correia, Jakob Manthey, Anastasia Månsson, Peter Allebeck, Ludwig Kraus, Jürgen Rehm

Contents

[Appendix 1: Overview of major alcohol control policies 2](#_Toc215843857)

[Appendix 2: The BtG-M scale 5](#_Toc215843858)

[Appendix 3: Sample description 8](#_Toc215843859)

[Appendix 4: Step-by-step construction of the main model for the primary outcome 10](#_Toc215843860)

[Appendix 5: Sensitivity and additional post-hoc analyses for the primary outcome 13](#_Toc215843861)

[Appendix 6: Results for secondary outcomes 17](#_Toc215843862)

[Appendix 7: Power analysis 19](#_Toc215843863)

# Appendix 1: Overview of major alcohol control policy changes

**Table A1.** Overview of major alcohol control policy changes in Estonia, France, Italy, Latvia, Lithuania, and Poland, between 2000 and 2020.

| Year | Estonia | France | Italy | Latvia | Lithuania | Poland |
| --- | --- | --- | --- | --- | --- | --- |
| 2000 |  | **Jun-22:** Licensing system for off-premise sales; restrictions on alcohol sales; limit of one on-site outlet per 450 inhabitants |  |  |  | **Jan-1:** Small increase in excise taxation  **Apr-13:** Small increase in excise taxation for beer only |
| 2001 | **Feb-1**: BAC limit of 0.2 g/l for drivers |  | **Mar-30:** Ban on off-premises sales between midnight and 7 a.m.; spirits sales restrictions at petrol stations between 2 and 6 a.m.; restriction on spirits advertisements between 4 and 7 p.m. on radio and television and “youth protection”; BAC limit of 0.5 g/l introduced |  | **Nov-28:** Alcohol sales allowed at petrol stations | **Jan-1:** Small increase in excise taxation  **Jun-28:** Exemption for beer introduced in advertising ban |
| 2002 |  |  |  | **Jun-14:** Ban on off-premises sales between 10 p.m. and 8 a.m.; alcohol ads banned at children's and youth sports events |  | **Oct-1:** Reduction in excise taxation by 30% |
| 2003 |  |  |  |  |  | **May-25:** Return of beer advertising on billboards; TV, radio, and theatre ad ban reduced to 6 a.m. to 8 p.m. |
| 2004 | **Apr-15:** Alcohol sales banned at kiosks and street/market trading |  |  | **Jan-1**: BAC limit set to 0.2 g/l for new drivers; 0.1 g/l for moped and bicycle drivers  **May-1:** Alcohol ads banned in printed press and on public transportation |  | **Jan-1:** Reduction in excise taxation for spirits |
| 2005 |  |  | **Mar-14:** Excise tax increase |  |  | **Jan-1:** Excise tax increase |
| 2006 |  |  |  | **Jul-1:** BAC limit set to 0.5 g/l for moped and bicycle drivers |  |  |
| 2007 |  |  | **Aug-3:** Ban on off-premises sales changed to midnight–6 a.m.; on-premises sales banned between 3 and 6 a.m.  **Oct-2:** On-premises sales ban changed to 2 to 6 a.m. |  | **Jan-1:** Excise tax increases, lowering affordability; year of sobriety (daytime alcohol ads banned on TV/radio) |  |
| 2008 | **Jan-1 / Jul-1:** Excise tax increase, lowering affordability **Jul-14:** Off-premises sales banned from 10 p.m. to 10 a.m.  **Nov-1:** Alcohol ads banned on broadcasting from 7 a.m. to 9 p.m. |  |  |  | **Jan-1:** Stricter penalties for drink-driving; BAC reduced to 0.2 g/l for novice drivers |  |
| 2009 |  | **Apr-10**: Excise tax increase  **Jul-23:** Minimum drinking age raised from 16 to 18; ban on refrigerated alcohol sales at fuel outlets |  | **Feb-1 /Jul-1:** Excise tax increases, lowering affordability | **Jan-1:** Ban on off-premises sales between 10 p.m. and 8 a.m. | **Jan-1:** Excise tax increase  **Mar-1:** Excise tax increases, lowering affordability |
| 2010 | **Jan-1:** Excise tax increases, lowering affordability | **May-1**: Excise tax increase | **Jul-29:** On-premises sales ban reverted to 3 a.m. to 6 a.m. | **Feb-1:** Excise tax increases, lowering affordability |  |  |
| 2011 |  | **Dec-23**: Excise tax increase on spirits; decrease on intermediate products |  |  |  |  |
| 2012 |  | **Dec-19**: Excise tax increase on beer | **Sep-13:** Increase minimum legal drinking purchasing age from 16 to 18 for all beverages | **Jan-1:** BAC limit set to 0.2 g/l for bus, tram, and trolley drivers |  |  |
| 2013 |  | **Jun-7**: Excise tax increase |  | **Jul-19:** Outdoor alcohol advertising banned |  |  |
| 2014 | **Jul-1:** Notification obligation replaces licensing | **Jan-1** and **May-30**: Excise tax increases | **Jan-1:** Excise tax increase |  |  | **Jun-4**: Excise tax increase |
| 2015 |  | **Jun-6**: Excise tax increase  **Jun-28:** 0.2 g/l BAC limit for novice and professional drivers | **Jan-1:** Excise tax increase |  | **Jan-1:** BAC limit set to 0.0 g/l for professional and motorbike drivers |  |
| 2016 | **Feb-1:** Excise tax increases, lowering affordability | **Jan-1** and **Jun-13**: Excise tax increases |  |  | **Jan-1:** Alcohol sales banned at petrol stations |  |
| 2017 | **Feb-1 / Jul-1:** Excise tax increases, lowering affordability |  | **Jan-1:** Excise tax decrease on beer |  | **Mar-1:** Excise tax increases, lowering affordability |  |
| 2018 | **Feb-1:** Excise tax increases, lowering affordability **Jun-1:** Alcohol advertising ban extended (7 a.m. to 10 p.m.) | **Jun-23**: Excise tax increases |  |  | **Jan-1:** Off-premises sales hours reduced (10 a.m.–8 p.m. Mon-Sat, 10 a.m.–3 p.m. Sun); full ban on TV, radio, and internet alcohol ads; minimum legal purchasing age raised from 18 to 20 for all beverages |  |
| 2019 | **Jun-1:** Partial ban on point-of-sale alcohol displays  **Jul-1:** 25% reduction in excise tax | **Jun-8**: Excise tax increases | **Jan-1:** Excise tax decrease on beer | **Mar-1:** Excise tax increases, lowering affordability |  |  |
| 2020 |  | **Jul -25**: Excise tax increases |  |  |  | **Jan-1:** Excise tax increases, lowering affordability |

# Note: BAC, blood alcohol concentration.

# Appendix 2: The BtG-M scale

**Table A2.** BtG-M scale questionnaire (after Karlsson and Österberg, 2007)

| Policy subgroup | Answer/score |
| --- | --- |
| Control of production and wholesale of alcohol | |
| 1. State monopoly for the production or wholesale of: | - Beer (0.5 p) - Wine (0.5 p) - Spirits (1 p) |
|  | **Point total -/2** |
| Control of distribution | |
| 2.1. State monopoly for off-premise sale of: | - Beer (1 p) - Wine (1 p) - Spirits (2 p) |
| 2.2. No state monopoly, but restrictive license system for off-premise sales of: | - Beer (0.5 p) - Wine (0.5 p) - Spirits (1 p) |
| 2.3. Special permanent restrictions on (in *off-premise* sales): | - Sale days (1 p) - Sale hours (1 p) |
| 2.4. Other special permanent restrictions on places of sale (in *off-premise* sales): | - Yes (1 p) |
| 2.5. Special permanent restrictions on (in *on-premise* sales): | - Sale days (1 p) - Sale hours (1 p) |
| 2.6. Other special permanent restrictions on places of sale (in *on-premise* sales): | - Yes (1 p) |
|  | **Point total -/10** |
| Personal Control | |
| 3.1. Legal age limit for *on-premise* sales at least: | - 16 for all alcoholic beverages (0.5 p) - 18 for some alcoholic beverages (1 p) - 18 for all alcoholic beverages (1.5 p) - 20 for some alcoholic beverages (2 p) |
| 3.2. Legal age limit for *off-premise* sales at least: | - 16 for all alcoholic beverages (0.5 p) - 18 for some alcoholic beverages (1 p) - 18 for all alcoholic beverages (1.5 p) - 20 for some alcoholic beverages (2 p) |
|  | **Point total -/4** |
| Control of marketing | |
| 4.1. Regulation on alcohol advertisement: | - Statutory control on national alcohol advertising for some alcoholic beverages (1 p) - Ban on national alcohol advertisement on some types of advertising media (1.5 p) - Ban on national alcohol advertisement for some types of alcoholic beverages (2 p) - Ban on all national alcohol advertising and sponsorship (3 p) |
|  | **Point total -/3** |
| Social and environmental controls | |
| 5.1. Drunk driving (BAC limit) | - 0.08% (1 p) - 0.05% (2 p) - <0.05% (4 p) |
|  | **Point total -/4** |
| Public policy | |
| 6.1. National alcohol prevention or education program | - Yes (1 p) |
|  | **Point total -/1** |
| Alcohol taxation | |
| 7.1. Excise duty on strong alcohol beverages (spirits) per hectolitre of 100% alcohol; in USD, PPP-adjusted | - $1 to $1249 (0.5 p) - $1250 to $2499 (1 p) - $2500 to $3749 (1.5 p) - $3750 to $4999 (2 p) - $5000 to $6249 (2.5 p) - $6250 to $7499 (3 p) - $7500 to $8749 (3.5 p) - ≥ $8050 (4 p) |
| 7.2 Excise duty on intermediate products, per hectolitre of 100% alcohol; in USD, PPP-adjusted | - $1 to $1249 (0.5 p) - $1250 to $2499 (1 p) - $2500 to $3749 (1.5 p) - $3750 to $4999 (2 p) - $5000 to $6249 (2.5 p) - $6250 to $7499 (3 p) - $7500 to $8749 (3.5 p) - ≥ $8050 (4 p) |
| 7.3. Excise duty on still wine, per hectolitre of 100% alcohol; in USD, PPP-adjusted | - $1 to $999 (0.5 p) - $1000 to $1999 (1 p) - $2000 to $2999 (1.5 p) - $3000 to $3999 (2 p) - $4000 to $4999 (2.5 p) - $5000 to $5999 (3 p) - $6000 to $6999 (3.5 p) - ≥ $7000 (4 p) |
| 7.4. Excise duty on beer, per hectolitre of 100% alcohol; in USD, PPP-adjusted | - $1 to $1124 (0.5 p) - $1125 to $2249 (1 p) - $2250 to $3374 (1.5 p) - $3375 to $4499 (2 p) - $4500 to $5624 (2.5 p) - $5625 to $6749 (3 p) - $6750 to $7874 (3.5 p) - ≥ $7875 (4 p) |
|  | **Total points -/16** |
| Total score | **-/40** |

Note: BAC, blood alcohol concentration.

**Table A3.** Partial BtG-M scores after rescaled each domain to range from 0 (least restrictive) to 1 (most restrictive), by country and year of ESPAD data collection wave.

|  | **Control of production** | **Control of distribution** | **Personal control** | **Control of marketing** | **Social and environmental controls** | **Public policy** | **Alcohol taxation** |
| --- | --- | --- | --- | --- | --- | --- | --- |
| Estonia |  |  |  |  |  |  |  |
| 2007 | - | 0.67 | 0.67 | 0.50 | 1.00 | 0.00 | 0.30 |
| 2011 | - | 0.67 | 0.67 | 0.00 | 1.00 | 0.00 | 0.50 |
| 2015 | - | 0.00 | 0.67 | 0.00 | 1.00 | 1.00 | 0.60 |
| 2019 | - | 0.00 | 0.67 | 0.25 | 1.00 | 1.00 | 1.00 |
| France |  |  |  |  |  |  |  |
| 2007 | - | 0.33 | 0.00 | 0.25 | 0.00 | 1.00 | 0.30 |
| 2011 | - | 0.33 | 0.67 | 0.25 | 0.00 | 1.00 | 0.30 |
| 2015 | - | 0.33 | 0.67 | 0.25 | 0.00 | 1.00 | 0.30 |
| 2019 | - | 0.33 | 0.67 | 0.25 | 0.00 | 1.00 | 0.30 |
| Italy |  |  |  |  |  |  |  |
| 2007 | - | 0.67 | 0.00 | 0.00 | 0.00 | 0.00 | 0.00 |
| 2011 | - | 1.00 | 0.00 | 0.00 | 0.00 | 0.00 | 0.00 |
| 2015 | - | 1.00 | 0.67 | 0.00 | 0.00 | 0.00 | 0.10 |
| 2019 | - | 1.00 | 0.67 | 0.00 | 0.00 | 0.00 | 0.20 |
| Latvia |  |  |  |  |  |  |  |
| 2007 | - | 0.33 | 0.67 | 0.50 | 0.00 | 1.00 | 0.20 |
| 2011 | - | 0.33 | 0.67 | 0.50 | 0.00 | 0.00 | 0.40 |
| 2015 | - | 0.33 | 0.67 | 0.50 | 0.00 | 0.00 | 0.40 |
| 2019 | - | 0.33 | 0.67 | 0.50 | 0.00 | 0.00 | 0.60 |
| Lithuania |  |  |  |  |  |  |  |
| 2007 | - | 0.33 | 0.67 | 0.00 | 1.00 | 1.00 | 0.20 |
| 2011 | - | 0.67 | 0.67 | 0.25 | 1.00 | 0.00 | 0.40 |
| 2015 | - | 1.00 | 0.67 | 0.25 | 1.00 | 1.00 | 0.50 |
| 2019 | - | 1.00 | 1.00 | 1.00 | 1.00 | 1.00 | 1.00 |
| Poland |  |  |  |  |  |  |  |
| 2007 | - | 0.33 | 0.67 | 0.50 | 1.00 | 1.00 | 0.20 |
| 2011 | - | 0.33 | 0.67 | 0.50 | 1.00 | 1.00 | 0.30 |
| 2015 | - | 0.33 | 0.67 | 0.50 | 1.00 | 1.00 | 0.30 |
| 2019 | - | 0.33 | 0.67 | 0.50 | 1.00 | 1.00 | 0.40 |

# Appendix 3: Sample description

**Table A4**. Individual-level characteristics, by country and year of data collection.

|  | **2007 (n=21 624)** | **2011 (n=20 605)** | **2015 (n=24 373)** | **2019 (n=17 587)** | **Total (n=84 189)** |
| --- | --- | --- | --- | --- | --- |
| Country, n (%) |  |  |  |  |  |
| Lithuania | 2345 (10.8) | 2405 (11.7) | 2518 (10.3) | 2372 (13.5) | 9640 (11.5) |
| Estonia | 2330 (10.8) | 2413 (11.7) | 2419 (9.9) | 2478 (14.1) | 9640 (11.5) |
| France | 2882 (13.3) | 2549 (12.4) | 2674 (11.0) | 2539 (14.4) | 10 644 (12.6) |
| Italy | 9762 (45.1) | 4807 (23.3) | 4016 (16.5) | 2510 (14.3) | 21 095 (25.1) |
| Latvia | 2208 (10.2) | 2559 (12.4) | 1044 (4.3) | 2689 (15.3) | 8500 (10.1) |
| Poland | 2097 (9.7) | 5872 (28.5) | 11 702 (48.0) | 4999 (28.4) | 24 670 (29.3) |
| Sex, n (%) |  |  |  |  |  |
| Male | 11 008 (50.9) | 10 104 (49.0) | 11 963 (49.1) | 8588 (48.8) | 41 663 (49.5) |
| Female | 10 616 (49.1) | 10 501 (51.0) | 12 410 (50.9) | 8999 (51.2) | 42 526 (50.5) |
| *Social Behaviours* |  |  |  |  |  |
| Participating in sports, n (%) |  |  |  |  |  |
| Less than once or twice a month | 5660 (26.2) | 4091 (19.9) | 4291 (17.6) | 3480 (19.8) | 17 522 (20.8) |
| At least once a week | 15 848 (73.3) | 16 385 (79.5) | 19 948 (81.8) | 14 011 (79.7) | 66 192 (78.6) |
| Missing | 116 (0.5) | 129 (0.6) | 134 (0.5) | 96 (0.5) | 475 (0.6) |
| Going out in the evening, n (%) |  |  |  |  |  |
| Less than once or twice a month | 9563 (44.2) | 9953 (48.3) | 14 009 (57.5) | 10 749 (61.1) | 44 274 (52.6) |
| At least once a week | 11 898 (55.0) | 10 437 (50.7) | 10 163 (41.7) | 6723 (38.2) | 39 221 (46.6) |
| Missing | 163 (0.8) | 215 (1.0) | 201 (0.8) | 115 (0.7) | 694 (0.8) |
| Hanging out with friends, n (%) |  |  |  |  |  |
| Less than once or twice a month | 6060 (28.0) | 6555 (31.8) | 9716 (39.9) | 7424 (42.2) | 29 755 (35.3) |
| At least once a week | 15 462 (71.5) | 13 944 (67.7) | 14 515 (59.6) | 10 083 (57.3) | 54 004 (64.1) |
| Missing | 102 (0.5) | 106 (0.5) | 142 (0.6) | 80 (0.5) | 430 (0.5) |
| Using the Internet for leisure, n (%) |  |  |  |  |  |
| Less than once or twice a month | 5603 (25.9) | 1591 (7.7) | 1577 (6.5) | 709 (4.0) | 9480 (11.3) |
| At least once a week | 15 900 (73.5) | 18 883 (91.6) | 22 644 (92.9) | 16 797 (95.5) | 74 224 (88.2) |
| Missing | 121 (0.6) | 131 (0.6) | 152 (0.6) | 81 (0.5) | 485 (0.6) |
| *Access to alcoholic beverages* |  |  |  |  |  |
| Beer, n (%) |  |  |  |  |  |
| Impossible or very or fairly difficult | 3154 (14.6) | 3098 (15.0) | 4681 (19.2) | 3735 (21.2) | 14 668 (17.4) |
| Fairly or very easy | 16 675 (77.1) | 15 701 (76.2) | 17 292 (70.9) | 11 967 (68.0) | 61 635 (73.2) |
| Missing | 1795 (8.3) | 1806 (8.8) | 2400 (9.8) | 1885 (10.7) | 7886 (9.4) |
| Wine, n (%) |  |  |  |  |  |
| Impossible or very or fairly difficult | 4582 (21.2) | 4468 (21.7) | 6491 (26.6) | 4997 (28.4) | 20 538 (24.4) |
| Fairly or very easy | 14 593 (67.5) | 13 800 (67.0) | 14 555 (59.7) | 10 275 (58.4) | 53 223 (63.2) |
| Missing | 2449 (11.3) | 2337 (11.3) | 3327 (13.7) | 2315 (13.2) | 10 428 (12.4) |
| Spirits, n (%) |  |  |  |  |  |
| Impossible or very or fairly difficult | 6885 (31.8) | 6695 (32.5) | 8760 (35.9) | 6575 (37.4) | 28 915 (34.3) |
| Fairly or very easy | 12 066 (55.8) | 11 513 (55.9) | 12 450 (51.1) | 8756 (49.8) | 44 785 (53.2) |
| Missing | 2673 (12.4) | 2397 (11.6) | 3163 (13.0) | 2256 (12.8) | 10 489 (12.5) |


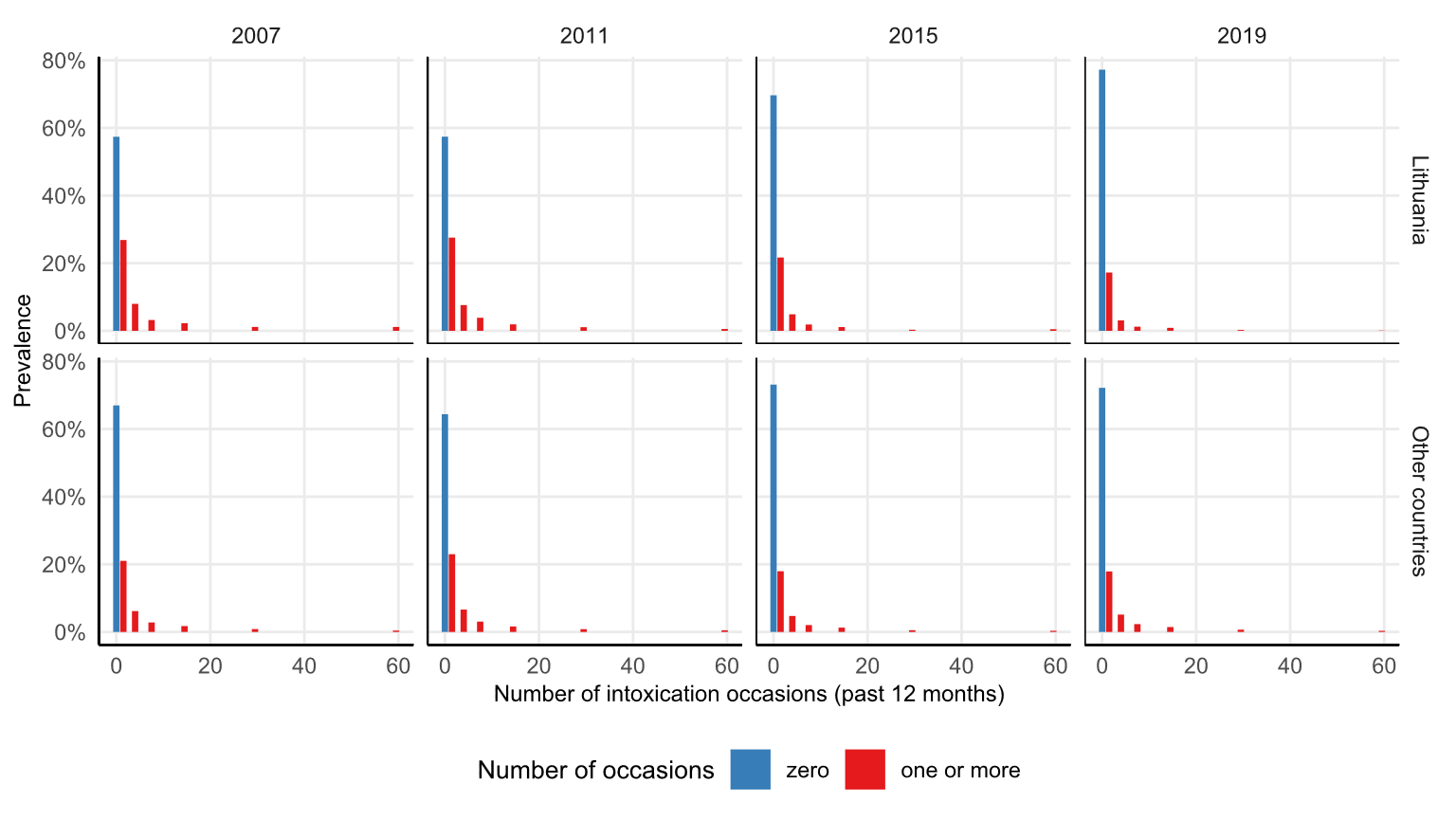


**Figure A1.** Distribution of frequency of intoxication in the past 12 months among adolescents, comparing Lithuania with the other five countries in the study, by year of data collection.

**Table A5.** Observed adolescent intoxication in the past 12 months, total, by country and year of data collection.

|  | **Frequency of intoxication,**  **mean (SD)** | **Prevalence of intoxication,**  **% (n)** | **Frequency of intoxication among those reporting intoxication,**  **mean (SD)** |
| --- | --- | --- | --- |
| Total | 1.4 (4.98) | 31.2% (26 267) | 4.48 (8.1) |
| Country |  |  |  |
| Lithuania | 1.54 (5.44) | 34.5% (3327) | 4.45 (8.54) |
| Estonia | 1.15 (3.91) | 33.3% (3211) | 3.47 (6.16) |
| France | 1.58 (4.94) | 34.2% (3643) | 4.6 0(7.57) |
| Italy | 1.27 (4.70) | 27.3% (5766) | 4.63 (8.09) |
| Latvia | 1.82 (5.96) | 38.9% (3308) | 4.67 (8.83) |
| Poland | 1.33 (5.02) | 28.4% (7012) | 4.67 (8.55) |
| ESPAD collection wave |  |  |  |
| 2007 | 1.6 (5.41) | 34.0% (7361) | 4.71 (8.45) |
| 2011 | 1.64 (5.38) | 36.4% (7503) | 4.51 (8.16) |
| 2015 | 1.16 (4.54) | 27.2% (6633) | 4.25 (7.90) |
| 2019 | 1.18 (4.46) | 27.1% (4770) | 4.36 (7.71) |

# Appendix 4: Step-by-step construction of the main model for the primary outcome


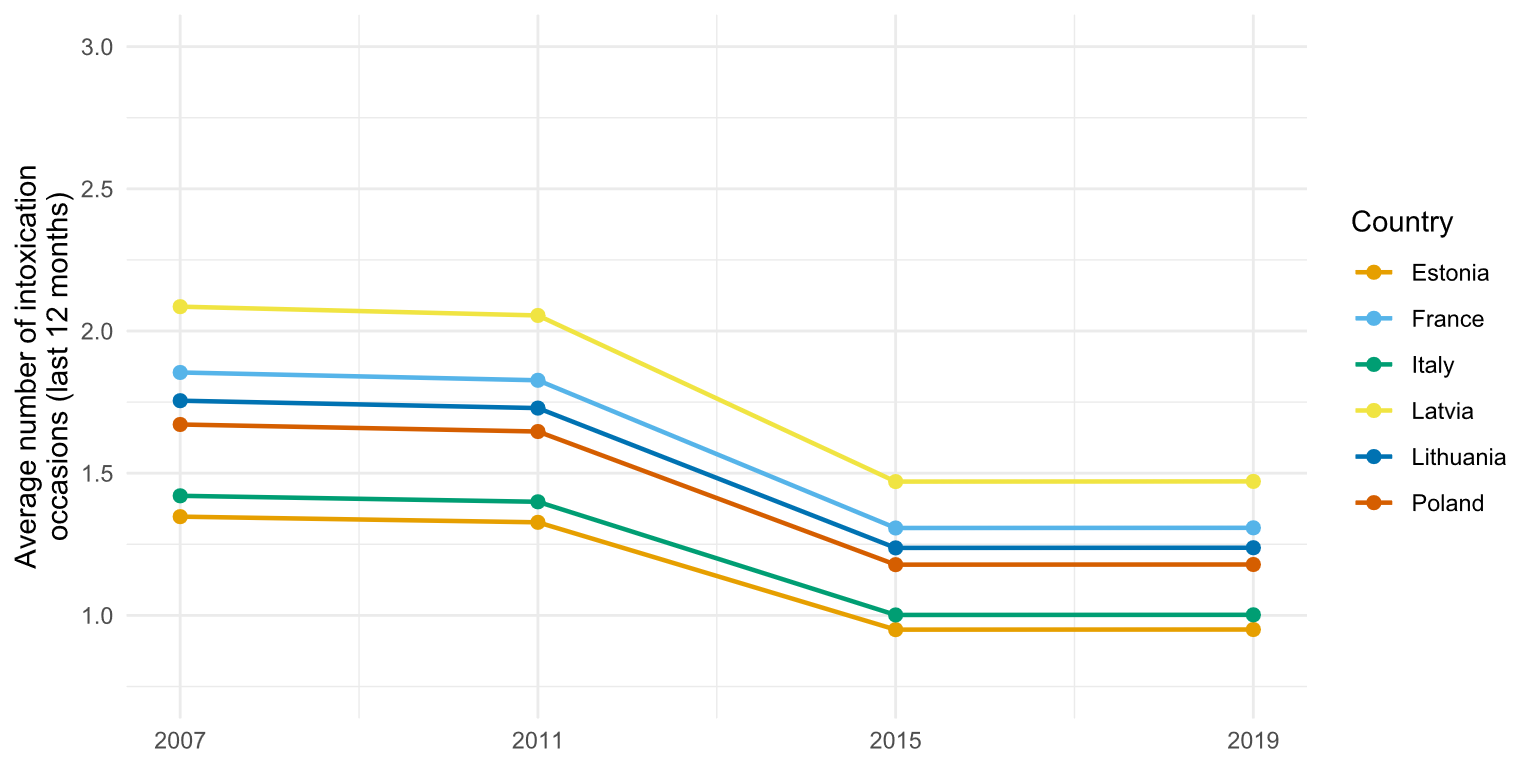


**Figure A2.** Predicted mean frequency of adolescent intoxication by country and year, adjusted for secular trend.


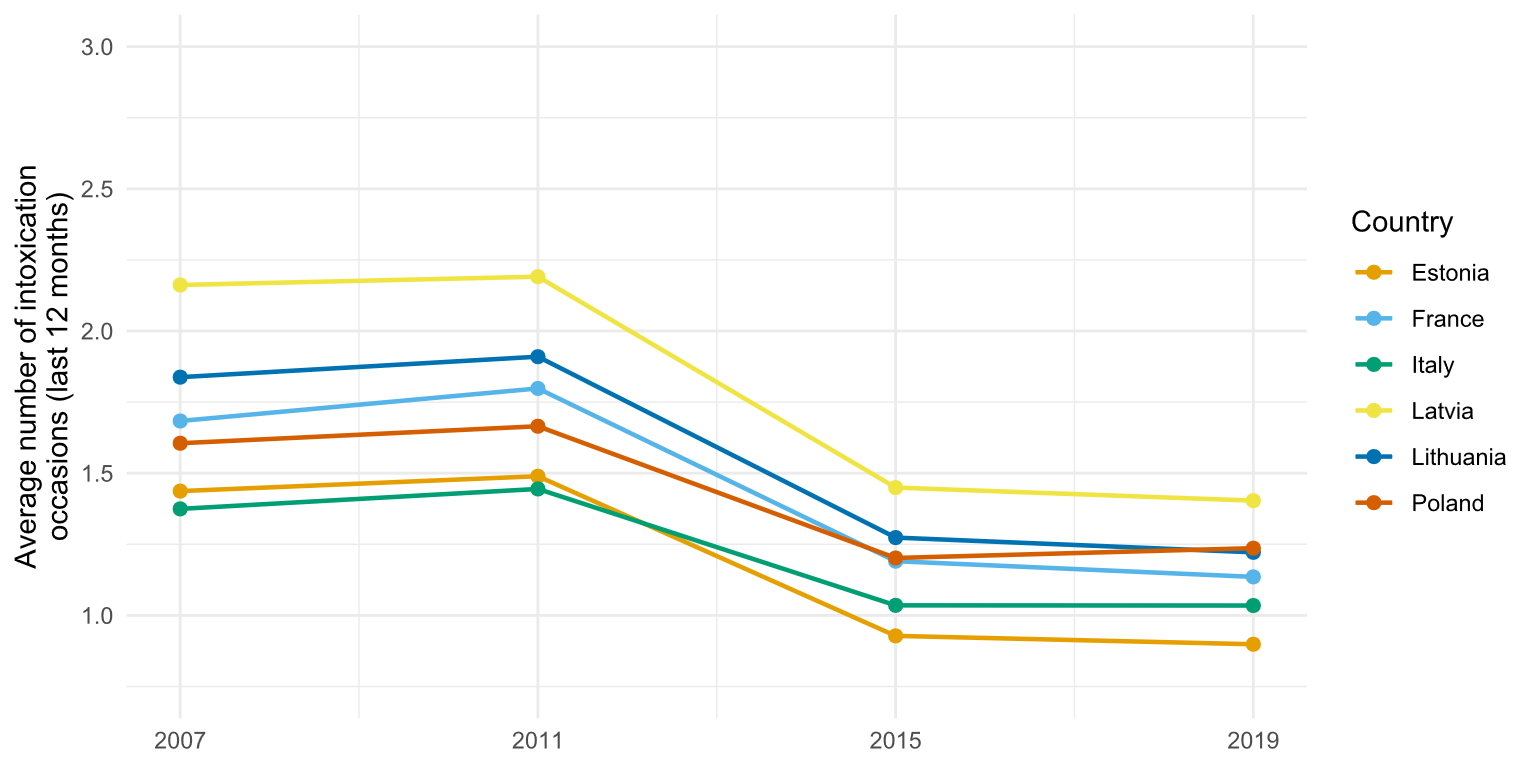


**Figure A3.** Predicted mean frequency of adolescent intoxication by country and year, adjusted for secular trend and individual-level differences (gender and social behaviours).


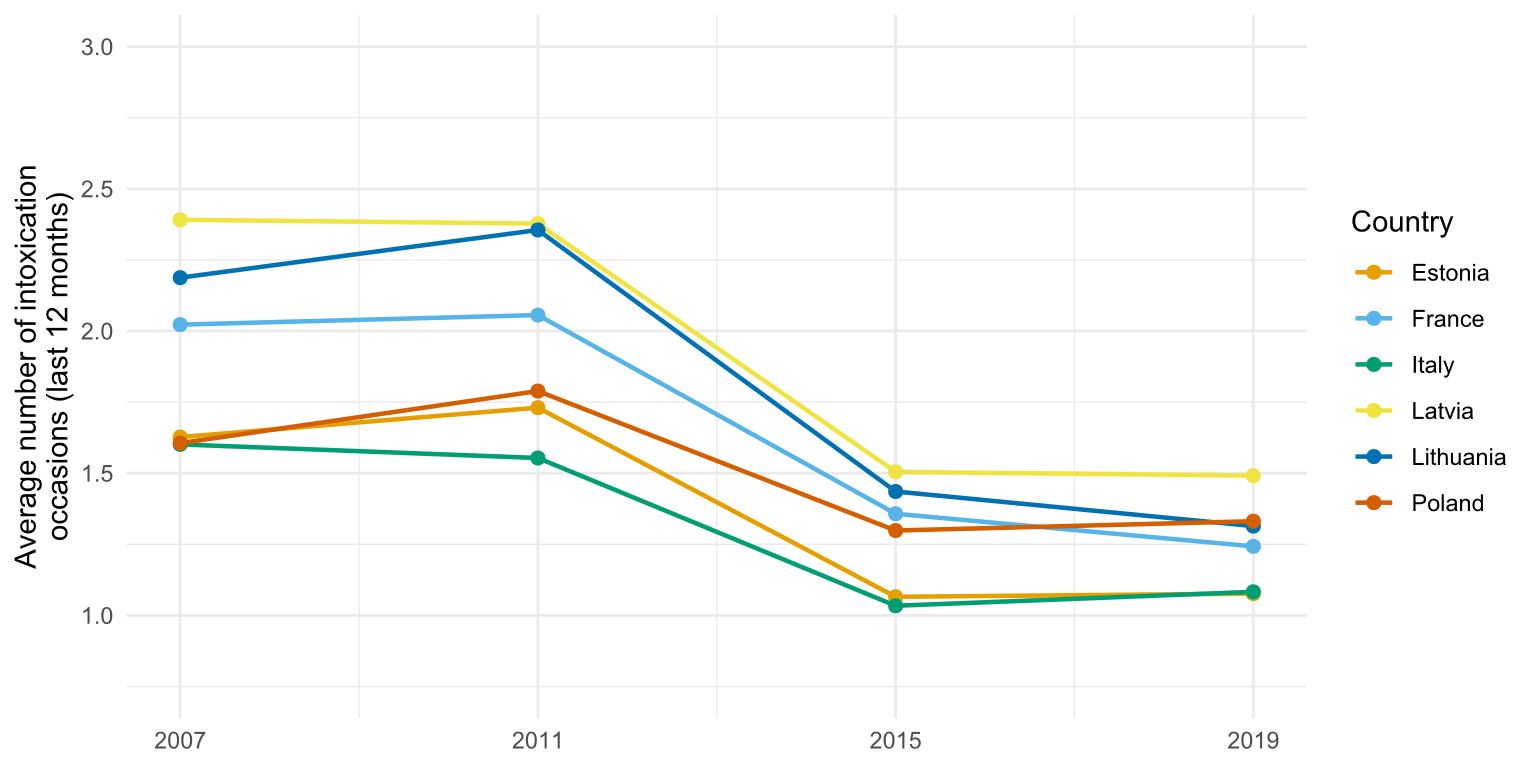


**Figure A4.** Predicted mean frequency of adolescent intoxication by country and year, adjusted for secular trend, individual-level differences (gender and social behaviours), and differences in self-perceived availability of alcoholic beverages.


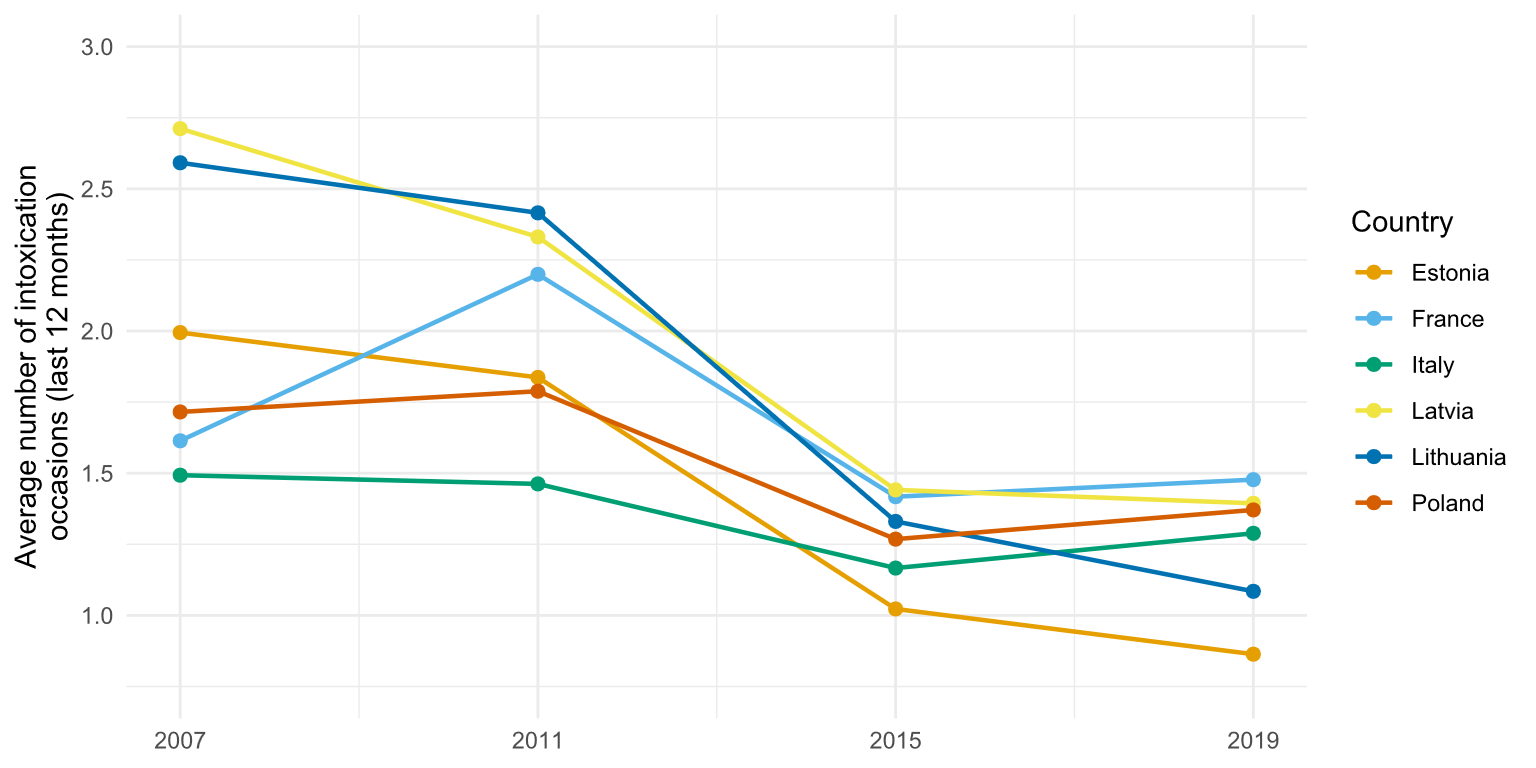


**Figure A5.** Predicted mean frequency of adolescent intoxication by country and year, adjusted for secular trend, individual-level differences (gender and social behaviours), differences in self-perceived availability of alcoholic beverages, and alcohol control policies other than marketing (taxation and personal control)


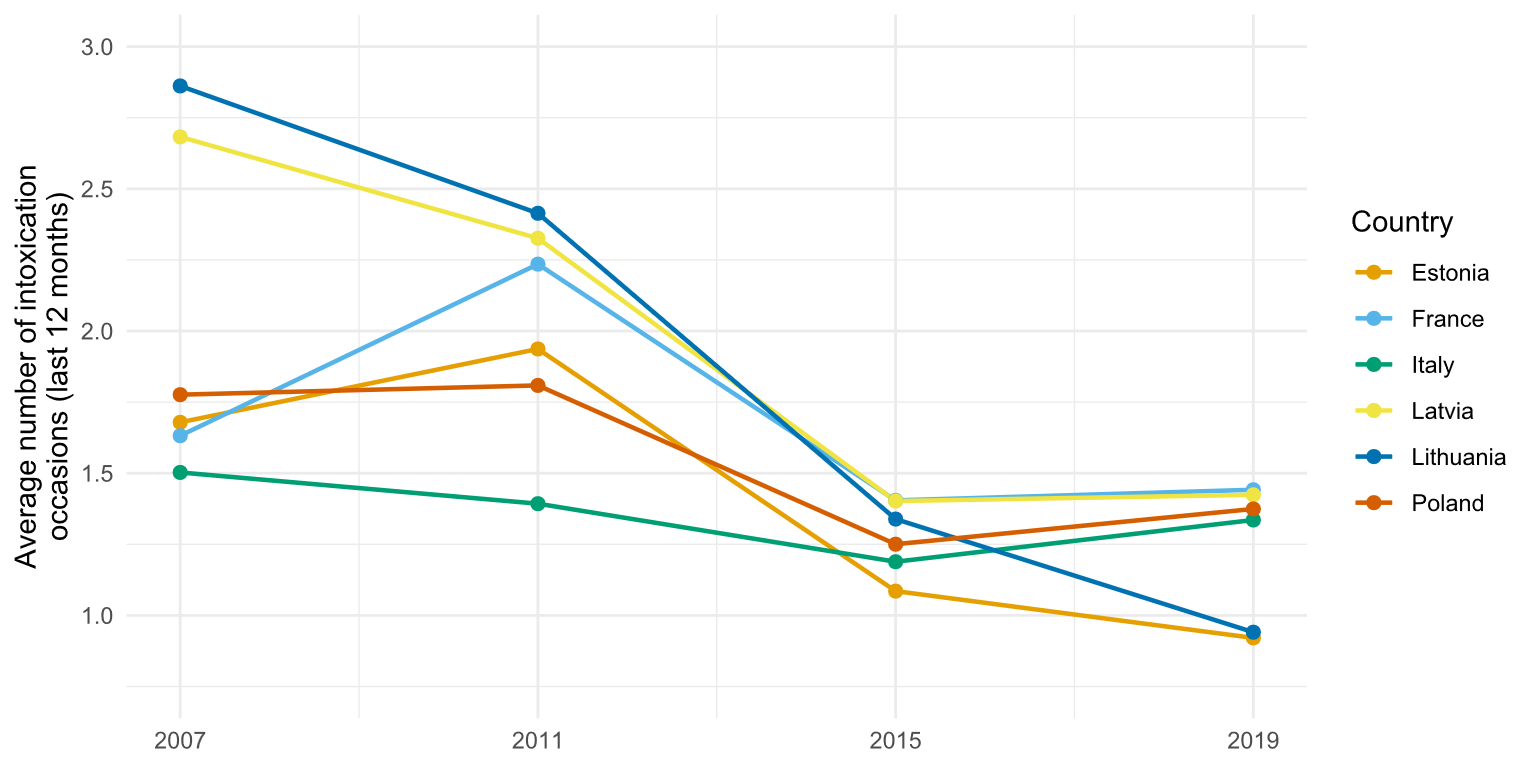


**Figure A6.** Predicted mean frequency of adolescent intoxication by country and year, adjusted for secular trend, individual-level differences (gender and social behaviours), differences in self-perceived availability of alcoholic beverages, alcohol control policies other than marketing (taxation and personal control), and alcohol marketing.

# Appendix 5: Sensitivity and additional post-hoc analyses for the primary outcome

**Figure A7.** Estimated impact of Lithuania’s 2018 alcohol marketing ban on prevalence of adolescent intoxication (two-part model), based on ESPAD data


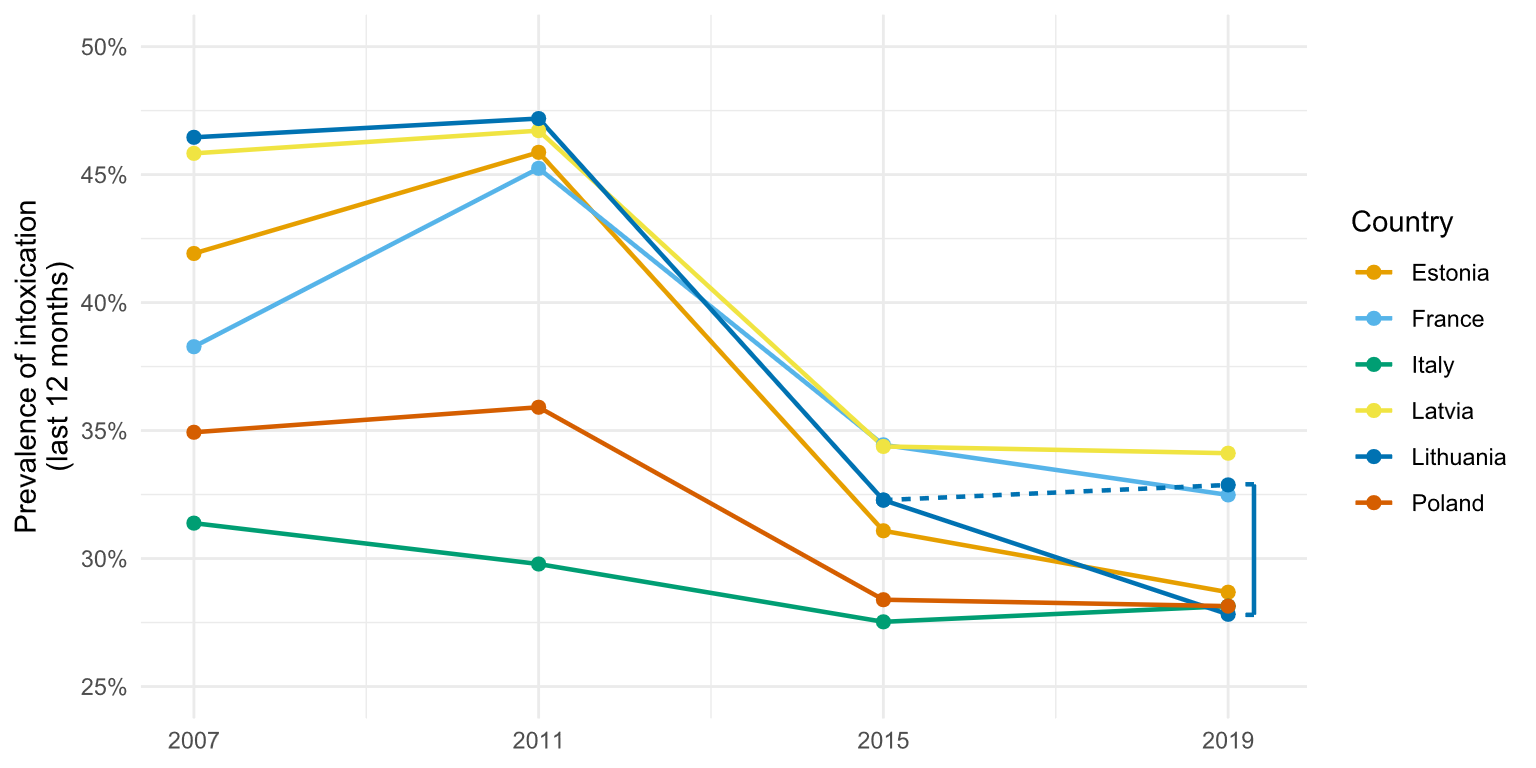


Note: Full lines represent the modelled values by the adjusted logistic regression model. Dashed line represents the predicted value for Lithuania if the 2018 alcohol marketing ban had not been implemented while keeping all other policy changes in effect. The square bracket indicates the impact of Lithuania’s 2018 alcohol marketing ban on frequency of intoxication.

**Table A6.** Adjusted regression models for assessing frequency of intoxication (main outcome) among adolescents in the ESPAD study, using a dummy variable contrasting a full national advertising and sponsorship ban (only in Lithuania in 2018) versus no such ban (sensitivity analysis)

|  | **Frequency of intoxication (n=84 189)** |
| --- | --- |
|  | **IRR (95%CI)*** |
| *BtG-M scores* |  |
| Personal control | 1.71 (1.51, 1.93) |
| Control of marketing (total ban on marketing) |  |
| No | Ref |
| Yes | 0.56 (0.46, 0.67) |
| Alcohol taxation | 0.76 (0.59, 0.98) |
| ESPAD collection wave |  |
| 2007 | Ref |
| 2011 | 1.01 (0.95, 1.08) |
| 2015 | 0.77 (0.71, 0.83) |
| 2019 | 0.83 (0.74, 0.92) |
| Sex |  |
| Male | Ref |
| Female | 0.70 (0.67, 0.72) |
| *Social Behaviours* |  |
| Sports participation |  |
| Less than once or twice a month | Ref |
| At least once a week | 0.71 (0.68, 0.74) |
| Going out in the evening |  |
| Less than once or twice a month | Ref |
| At least once a week | 2.36 (2.27, 2.45) |
| Hanging out with friends |  |
| Less than once or twice a month | Ref |
| At least once a week | 1.50 (1.44, 1.56) |
| Using the Internet for leisure |  |
| Less than once or twice a month | Ref |
| At least once a week | 0.90 (0.85, 0.96) |
| *Access to alcoholic beverages* |  |
| Beer |  |
| Impossible or very or fairly difficult | Ref |
| Fairly or very easy | 1.51 (1.41, 1.60) |
| Wine |  |
| Impossible or very or fairly difficult | Ref |
| Fairly or very easy | 1.15 (1.09, 1.22) |
| Spirits |  |
| Impossible or very or fairly difficult | Ref |
| Fairly or very easy | 2.74 (2.61, 2.87) |
| Country |  |
| Lithuania | Ref |
| Estonia | 0.85 (0.78, 0.93) |
| France | 1.16 (1.06, 1.27) |
| Italy | 0.59 (0.52, 0.68) |
| Latvia | 1.13 (1.04, 1.23) |
| Poland | 0.73 (0.67, 0.78) |

* IRR: incidence rate ratios and respective 95% confidence intervals, obtained from adjusted negative binomial regression models.

**Table A7.** Adjusted regression models for assessing frequency of intoxication (main outcome) among adolescents in the ESPAD study, without the inclusion of the personal control domain from the BtG-M scale (post-hoc analysis).

|  | **Frequency of intoxication (n=84 189)** |
| --- | --- |
|  | **IRR (95%CI)*** |
| *BtG-M scores* |  |
| Control of marketing | 0.79 (0.68, 0.92) |
| Alcohol taxation | 0.47 (0.38, 0.58) |
| ESPAD collection wave |  |
| 2007 | Ref |
| 2011 | 1.15 (1.09, 1.21) |
| 2015 | 0.95 (0.90, 1.01) |
| 2019 | 1.10 (1.01, 1.19) |
| Sex |  |
| Male | Ref |
| Female | 0.69 (0.67, 0.72) |
| *Social Behaviours* |  |
| Sports participation |  |
| Less than once or twice a month | Ref |
| At least once a week | 0.71 (0.68, 0.74) |
| Going out in the evening |  |
| Less than once or twice a month | Ref |
| At least once a week | 2.35 (2.27, 2.44) |
| Hanging out with friends |  |
| Less than once or twice a month | Ref |
| At least once a week | 1.50 (1.44, 1.56) |
| Using the Internet for leisure |  |
| Less than once or twice a month | Ref |
| At least once a week | 0.90 (0.85, 0.96) |
| *Access to alcoholic beverages* |  |
| Beer |  |
| Impossible or very or fairly difficult | Ref |
| Fairly or very easy | 1.52 (1.43, 1.62) |
| Wine |  |
| Impossible or very or fairly difficult | Ref |
| Fairly or very easy | 1.16 (1.09, 1.22) |
| Spirits |  |
| Impossible or very or fairly difficult | Ref |
| Fairly or very easy | 2.69 (2.57, 2.82) |
| Country |  |
| Lithuania | Ref |
| Estonia | 0.94 (0.86, 1.02) |
| France | 1.01 (0.93, 1.10) |
| Italy | 0.39 (0.35, 0.43) |
| Latvia | 1.23 (1.13, 1.33) |
| Poland | 0.72 (0.66, 0.79) |

* IRR: incidence rate ratios and respective 95% confidence intervals, obtained from adjusted negative binomial regression models.

# Appendix 6: Results for secondary outcomes

**Table A8.** Adjusted regression models for assessing frequency of alcohol consumption in the past 12 months and frequency of binge drinking in the past 30 days, among adolescents in the ESPAD study.

|  | **Frequency of consumption (n=83 014)** | **Frequency of binge drinking (n=83 877)** |
| --- | --- | --- |
|  | **IRR (95%CI)*** | **IRR (95%CI)*** |
| Country |  |  |
| Lithuania | Ref | Ref |
| Estonia | 1.32 (1.25, 1.40) | 2.43 (2.27, 2.60) |
| France | 1.78 (1.66, 1.90) | 1.78 (1.67, 1.91) |
| Italy | 0.86 (0.80, 0.92) | 0.85 (0.79, 0.91) |
| Latvia | 1.28 (1.18, 1.38) | 1.91 (1.77, 2.07) |
| Poland | 0.97 (0.90, 1.06) | 1.31 (1.21, 1.41) |
| ESPAD collection wave |  |  |
| 2007 | Ref | Ref |
| 2011 | 0.99 (0.95, 1.03) | 0.87 (0.83, 0.90) |
| 2015 | 0.75 (0.71, 0.78) | 0.84 (0.81, 0.88) |
| 2019 | 0.87 (0.81, 0.93) | 0.81 (0.78, 0.85) |
| Sex |  |  |
| Male | Ref | Ref |
| Female | 0.76 (0.74, 0.77) | 0.72 (0.70, 0.74) |
| *Social Behaviours* |  |  |
| Sports participation |  |  |
| Less than once or twice a month | Ref | Ref |
| At least once a week | 0.94 (0.92, 0.96) | 0.86 (0.83, 0.89) |
| Going out in the evening |  |  |
| Less than once or twice a month | Ref | Ref |
| At least once a week | 1.84 (1.80, 1.88) | 2.07 (2.00, 2.13) |
| Hanging out with friends |  |  |
| Less than once or twice a month | Ref | Ref |
| At least once a week | 1.31 (1.28, 1.34) | 1.31 (1.27, 1.36) |
| Using the Internet for leisure |  |  |
| Less than once or twice a month | Ref | - |
| At least once a week | 1.11 (1.07, 1.15) | - |
| *Access to alcoholic beverages* |  |  |
| Beer |  |  |
| Impossible or very or fairly difficult | Ref | Ref |
| Fairly or very easy | 1.94 (1.87, 2.00) | 1.61 (1.53, 1.69) |
| Wine |  |  |
| Impossible or very or fairly difficult | Ref | Ref |
| Fairly or very easy | 1.33 (1.29, 1.37) | 1.12 (1.07, 1.17) |
| Spirits |  |  |
| Impossible or very or fairly difficult | Ref | Ref |
| Fairly or very easy | 1.78 (1.73, 1.83) | 2.08 (2.00, 2.16) |
| *BtG-M scores* |  |  |
| Control of distribution | 0.82 (0.75, 0.89) | 1.35 (1.23, 1.50) |
| Personal control | 1.48 (1.38, 1.59) | - |
| Control of marketing | 0.88 (0.79, 0.99) | 0.82 (0.72, 0.92) |
| Public policy | 0.89 (0.85, 0.93) | - |
| Alcohol taxation | 0.46 (0.40, 0.54) | - |

* IRR: incidence rate ratios and respective 95% confidence intervals, obtained from adjusted negative binomial regression models.

# Appendix 7: Power analysis

Previous multi-country studies examining the effects of alcohol marketing restrictions on population-level consumption have found only marginal or borderline-significant associations,^1,2^ suggesting the need for large dataset. Thus, to maximise sensitivity, we used the full available sample from six countries included in this study, across the four time points (2007, 2011, 2015, 2019), providing a total sample size of 84 189 adolescents.

To assess whether the sample size was sufficient to detect the observed effect of alcohol marketing restrictiveness on adolescent intoxication, we conducted a post-hoc power analysis based on our main model. The outcome was the frequency of alcohol intoxication in the past 12 months, analysed as a count variable using negative binomial regression. We performed n=1000 simulations using the following parameters:

- Sample size: 84 189
- Dispersion parameter (θ): 0.246 (from the fitted model)
- Observed effect size: IRR = 0.65 (log IRR = -0.431)
- Baseline mean frequency of intoxication: 1.40
- Alpha: 0.05 (two-sided)

The simulated power exceeded 99%, indicating that the study was well-powered to detect the observed effect size. All analyses were conducted in R version 4.3.3 using base functions and the MASS package. The R code used for this power analysis is available in the supplementary materials.

**References**

1 Nelson JP. Alcohol advertising bans, consumption and control policies in seventeen OECD countries, 1975–2000. *Appl Econ* 2010; **42**: 803–23.

2 Saffer H, Dave D. Alcohol consumption and alcohol advertising bans. *Appl Econ* 2002; **34**. DOI:10.1080/00036840110102743.
